# Lettuce-avicularin treatment reverses insulin resistance through stimulation of glycolytic kinases and insulin signaling molecules

**DOI:** 10.22038/ijbms.2020.49770.11368

**Published:** 2021-02

**Authors:** Joy A. Amadi, Peter U. Amadi, Uche C. Njoku, Chioma L. Onitchi

**Affiliations:** 1 Department of Nutrition and Dietetics, Imo State University Owerri, Imo State, Nigeria; 2 Deparment of Biochemistry, Imo State University Owerri, Imo State, Nigeria; 3 Department of Biochemistry, University of Port Harcourt, Choba, Rivers State, Nigeria

**Keywords:** Avicularin, Glycolysis, Incretins, Insulin resistance, Insulin signaling, Lettuce

## Abstract

**Objective(s):**

In order to recommend a more effective approach to manage insulin resistance, we monitored the activities of glycolytic kinases, insulin signaling molecules, and incretin hormones and identified the possible targets related to the insulin-sensitizing effects of combined pharmacological and dietary intervention involving avicularin and lettuce.

**Materials and Methods:**

Insulin resistance was induced in rats with a fructose-rich diet and confirmed from baseline analysis of FBS (>250 mg/dl), insulin (>25 µIU/ml), and HOMA-IR (>10). For 12 weeks, the insulin-resistant rats were treated exclusively with 5000 mg/kg b.w avicularin (DAvi) or by dietary placement on lettuce (DLet) or a combination of both and compared with non-insulin resistant rats.

**Results:**

Avicularin reversed alterations in HbA1c and insulin levels. DLet produced no significant effect on the incretins GLP 1 (*P*=0.909) and GIP (*P*=0.990), but DAvi slightly stimulated GLP 1 but not GIP. A strong positive correlation was found between improved β-cell responsiveness and the insulin signaling molecules: Akt2 (r=0.7248), IRS 1 (r=0.5173), and PI3K (r=0.7892). Only the combined avicularin and lettuce reversed the Akt2 levels (*P*=0.728). The lettuce meal slightly stimulated PI3K but normalized IRS 1 while avicularin treatment slightly stimulated IRS 1 but restored the PI3K levels (*P*=0.815). The positive correlation between β-cell responsiveness and hexokinase (r=0.5959), PFK (r=0.6222), and PK (r=0.6591) activities were statistically significant. Alterations in glycolytic kinases were reversed by DLet and in combination with avicularin.

**Conclusion:**

A combined pharmacological and dietary approach with avicularin and lettuce is required to effectively reverse insulin resistance.

## Introduction

Post-translational modifications and protein-protein interactions are by far the dominant means of regulating insulin-mediated glucose translocation to tissues (1-3). Insulin-induced glucose translocation or uptake is achieved via a complex network of protein-protein interactions referred to as insulin signaling or transduction. Defects or dysregulation of this complex cascade is the underlying cause of impaired insulin sensitivity and metabolic syndromes (4, 5). The impairment of insulin sensitivity otherwise referred to as insulin resistance is simply a pathological state where tissues either partly or completely fail to respond to insulin in free circulation which results in elevated fasting insulin and blood sugar levels (6). At the cellular level, insulin resistance is described as poor insulin signaling strength from insulin receptor downstream to the end substrate of the cascade (7). Multiple defects in the insulin receptor downstream cascade especially the IRS/PI3K pathway have been linked to insulin resistance and currently represent a promising druggable target for antidiabetic agents (8). Though, several factors contribute to the onset and aggravation of insulin resistance, numerous studies have implicated the loss of function of insulin receptor substrates (IRS) as the mechanistic link to the dysregulation of insulin signaling (9-11). IRS are proteins localized at the membranes of insulin-bound/activated receptors that mediate insulin signaling by acting as molecular adaptors or scaffolds for downstream effectors and receptor kinases (12-14). It was reported (15) that knockout mice for IRS 2 developed obesity and diabetes while another study (16) showed that up-regulation of IRS mitigates obesity and diabetes by sustaining insulin signaling. Other insulin signaling molecules implicated in impaired insulin sensitivity are phosphoinositol 3 kinase (PI3K) and serine/ threonine kinase (Akt), also known as protein kinase B (PKB). During insulin resistance, the PI3K/Akt/GLUT4 signaling pathway becomes dysfunctional in many tissues involved in glucose homeostasis (17, 18). The activation of PI3K is critical to insulin-mediated glucose entry into the cells (9) because its downstream effect also initiates the activation of Akt. Akt2 expressed majorly in insulin-sensitive tissues is activated via a PI3K dependent mechanism (19). Akt2 activation redistributes GLUT 4 from storage vesicles to the plasma membrane where it facilitates glucose entry into the cell (20). Several studies have linked insulin resistance to a dysfunctional Akt2 where its secretion is less than 50% compared with non-insulin resistant conditions (21). In obese subjects too, Akt2 secretion is extensively suppressed (22). Akt-signaling also facilitates cellular entry and utilization of glucose via a glycolysis-dependent mechanism. Primarily, phosphofructokinase is activated by Akt-signalling-mediated dephosphorylation of the phosphorylated phosphofructokinase thereby promoting glycolysis (23). Recent clinical trials involving participants from diverse ethnicities linked the down-regulation of glycolytic enzymes to insulin resistance (24-26). Therefore, effector molecules and dietary interventions capable of stimulating glycolytic kinases during insulin resistance are presently under investigation (27). Avicularin is a non-toxic quercetin flavonol with diverse medicinal properties including hepatoprotective, antioxidation, and anti-inflammatory effects (28). Avicularin possesses potent anti-obesity effects, promotes cellular uptake of glucose (29), and effectively inhibits α-glucosidase (30). Also, plant extracts rich in avicularin have been shown to enhance insulin sensitivity (31). However, no report to date has specifically attributed any insulin-sensitizing effects of such plants to avicularin. In addition to synthetic drugs, dietary interventions especially with vegetables are recommended for the management of insulin resistance (32-34). Lettuce is a widespread vegetable of the *Asteraceae* family, heavily consumed due to its nutritional and health benefits (35). Lettuce plants and their varieties are rich in flavonols (14, 15) which are central to its therapeutic effects. A variety of lettuce, the Rutgers Scarlet lettuce, reportedly produced anti-obesity and anti-diabetic effects in mouse models (36, 37). Other studies have shown that the dietary inclusion of lettuce improves both cholesterol and glucose homeostasis (38, 39). While these reports on both avicularin and lettuce strongly suggest a possible improvement during insulin-resistant conditions, no study to date has either investigated the effects of lettuce or avicularin during insulin resistant conditions. Two distinct groups from our lab separately investigated the insulin-sensitizing effects of either avicularin or lettuce on the activities of insulin signaling molecules, incretins, and the kinases that regulate glycolysis, and found varied distinct mechanisms. With that, we thus decided to apply a combined pharmacological and dietary approach with avicularin and lettuce to manage insulin resistance induced by a fructose-rich chow. 

## Materials and Methods

***Handling***

Forty-eight (48) adult male Wistar rats weighing 110–120g were acclimatized for 5 days before grouping. The study was carried out at Imo State University Owerri for 12 weeks, with protocol approval in line with the Guidelines for Handling of Laboratory Animals and Care (NIH Publication No. 85-23) as approved by the Biochemistry Research and Ethics Committee (number IMSU/BCM/ETS/20190415). During acclimatization, the rats were maintained *ad libitum *on normal rat feed pellets (UAC Nigeria Grand Cereals Vital Feeds, Jos, Plateau State, Nigeria) and water. The lettuce used for this study (*Lactuca sativa*), was fresh lettuce leaves chopped into bits similar to rat pellet-sized feeds. Avicularin was purchased from Sigma Aldrich St. Louis, MO, USA. LD50 was estimated with rats and confirmed at > 5000 mg/kg b.w (40). Avicularin was administered by oral gavaging while lettuce was pulverized and administered *ad libitum* through dietary inclusion in 80%:20% w/w mixture with the normal commercial rat feed (Table 1).

***Induction of insulin resistance***

Insulin resistance was induced in thirty-two (32) randomly selected rats. The high-fructose diet method was performed for induction of insulin resistance (41, 42). Briefly, the rats were placed on a Harlan Teklad, TD. 89247 60% fructose-rich chow *ad libitum* for 6 weeks. Insulin resistance was confirmed based on baseline analysis of FBS (>250 mg/dl), insulin (>25 µIU/ml), and HOMA-IR (>10), and the blood samples obtained to confirm this (before treatment) were collected via tail puncture. 

***Grouping, and sample collection***

The rats were divided into 6 groups of 8 rats as follows; control group without induced insulin resistance placed on the rat feeds and water (NC), and those placed on 80% w/w lettuce diet (CL), insulin-resistant rats untreated (DU), and those treated with either 5000 mg/kg b.w. avicularin (DAvi) or 80% w/w lettuce diet (DLet), or in combination of 5000 mg/kg b.w. avicularin, and 80% w/w lettuce diet (DAviLet). At the completion of the 12 weeks of feeding, all rats were anesthetized on diethyl ether after an overnight fast. Blood samples after sacrifice were taken via cardiac puncture. Blood samples were used for FBS and HbA1c analysis, and plasma for insulin, C-peptide, glucagon, and incretins. Enzyme assays, IRS, Akt2, and PI3K, were performed with homogenates from supernatants of the excised liver. The homogenates were prepared by immersing the whole liver in 0.25M ice-cold sucrose and homogenated with a Teflon Potter homogenizer (DWK Life Sciences, Wheaton, IL, USA) and afterward, centrifuged for 10 min at 10^4^ Rpm (43). 

***Sample analysis***

BMI was estimated from the ratio of weight (g) and the body length^2^ (cm^2^). FBS was estimated with an Accu-Chek® Meter (Roche Diabetes Care, USA) while the analysis of HbA1c was done with a Siemens/Bayer DCA 2000+ Analyzer. Insulin and C-peptide activities were assayed by ELISA with a Sigma Aldrich rats Insulin Elisa kit (St. Louis, MO USA) and a MyBiosource Inc. rat C-peptide Elisa kit (MyBiosource Inc. USA). Homeostasis Model of Assessment of insulin resistance (HOMA-IR) and fasting β-cell responsiveness (M0) were calculated as follows (44, 45); 

HOMA-IR=(glucose (mmol/l) * insulin (µU/ml)/22.5), where 1 mg/dl equals 0.055 mmol/l. 

M0=100×fasting C-peptide (µg/l)/fasting glucose concentration (mg/dl), where 1 ng/ml = 1 µg/l.

Glucagon assay and incretin hormones, GLP 1 and GIP, assay were carried out by ELISA with their respective Elisa kits from Sigma Aldrich St. Louis, MO USA. For the insulin signaling molecules, Akt2 was assayed with an ABCAM (ab208986) ELISA kit (ABCAM Plc UK) while IRs 1 and PI3K were assayed with their respective ELISA kits from MyBioSource Inc, USA. Hexokinase (rat HK ELISA kit), phosphofructokinase (rat PFK ELISA kit) and pyruvate kinase (rat PK ELISA kit) assay were performed with kits from MyBioSource Inc, USA. All ELISA assay protocols carried out in this study were exactly as described in their respective kit manuals.

***Statistical analysis***

A test of normality based on the Shapiro Wilk’s test (with SPSS v 20 statistical software) was performed to confirm the normality of distribution. The data were presented as means±standard deviations using the SPSS software. The mean differences using the least standard deviations among the groups were compared at 95% confidence interval with a One-way ANOVA tool of the SPSS software. Pearson’s correlation based on the GraphPad Prism v 7.04 was done to determine the relationship between the β-cell responsiveness of the rats and each biochemical parameter measured as 95% confidence interval. The graphical representations were also constructed with the GraphPad Prism.

## Results

**Figure 1:**
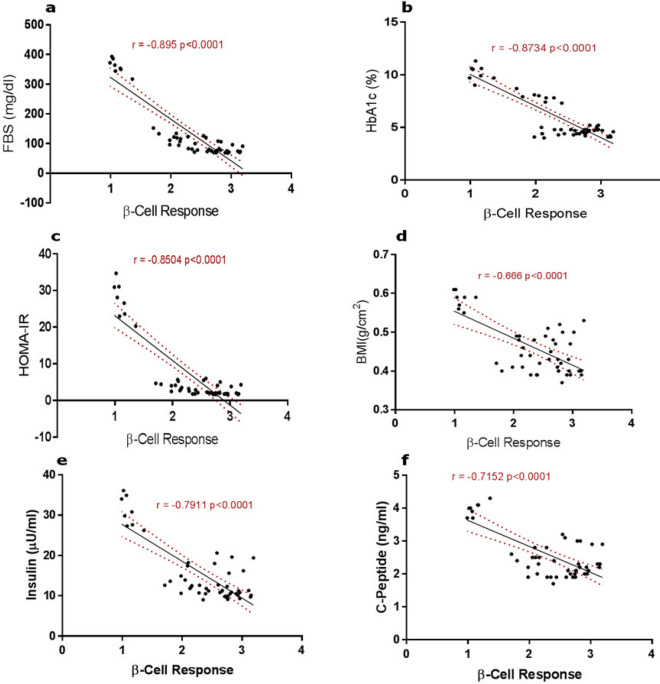
Correlation graphs of a) β-Cell response vs fasting blood sugar (FBS) b) β-Cell response vs glycated hemoglobin (HbA1c) c) β-Cell response vs homeostasis method of assessment of insulin resistance (HOMA-IR) d) β-Cell response vs body mass index (BMI) e) β-Cell response vs insulin activities f). β-Cell response vs c-peptide activities

The scatter plots in Figures 1a-f demonstrate the relationships between the β-cell responsiveness and FBS (Figure 1a), HbA1c (Figure 1b), HOMA-IR (Figure 1c), BMI (Figure 1d), insulin (Figure 1e), and c-peptide (Figure 1d) levels of rats under varying conditions of normoglycemia or insulin resistance and with various treatment approaches. A strong negative correlation was observed between increased β-cell response and FBS (r=-0.895), Hb1Ac (r=-0.8734), HOMA-IR (r = -0.8504), and BMI (r=-0.666). Also, the regression observed in the insulin (r=-0.7911) and c-peptide (r = -0.7152) activities, following increased β-cell response, was statistically significant (*P<*0.0001). 

Relative to the normoglycemic control, rats fed high fructose-diets showed significantly elevated (*P<*0.005) mean BMI values even when exclusively treated with avicularin (Table 2). The insulin-resistant rats exclusively fed on the lettuce meal or in combination with avicularin treatment showed BMI values within the control values (*P*=0.827 and 0.782, respectively). Rats treated with either avicularin or lettuce, or when combined, showed significantly decreased mean FBS levels (*P<*0.05). The exclusive avicularin-treatment to insulin-resistant rats produced Hb1Ac levels within the control values (*P*=0.995), as well as when combined with lettuce dietary inclusion (*P*=0.999). Insulin levels of the hyperinsulinemic rats after exclusive lettuce dietary inclusion were not significantly different from the control (*P*=0.078) and much decreased in combination with avicularin (*P*=0.867). Only the combinatorial treatment of the insulin-resistant rats with avicularin and lettuce produced c-peptide comparable to the normoglycemic rats. No significant difference was observed for the HOMA-IR values of the insulin-resistant rats placed on exclusive lettuce dietary treatment while avicularin administration significantly elevated the M0 levels comparably to the control. The combination of avicularin with a lettuce diet produced HOMA-IR and M0 levels within the normoglycemic range. In all parameters assessed, the exclusive treatment with either avicularin or lettuce produced significant changes in comparison with the control values.

**Figure 2:**
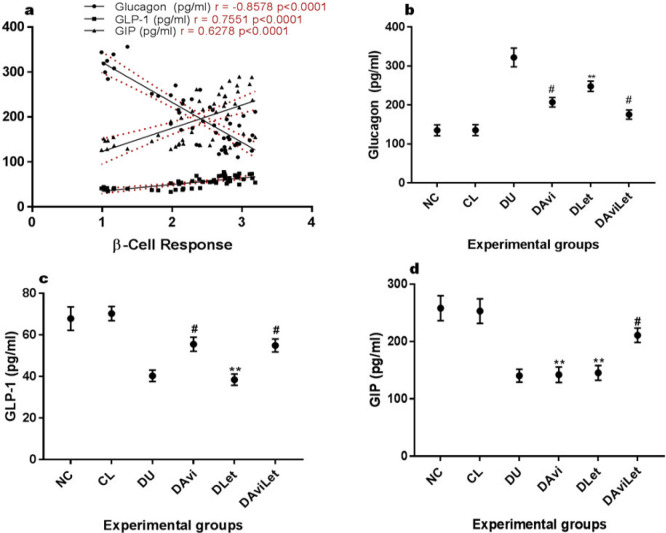
a) Correlation graph of β-Cell response vs glucagon, GLP 1, and GIP b) mean plasma glucagon levels c) mean plasma GLP 1 level d) mean plasma GIP levels. **- no significant changes relative to normoglycemic rats, #-significantly higher than the control but lower than the negative control (DU)

The relationships between β-cell responsiveness of the rats and the incretin hormones as well as glucagon were demonstrated in Figure 2a. As the β-cell responsiveness increased, the glucagon levels significantly decreased in a strong inverse relationship (r=-08578) while the scatter plot showed a strong positive correlation between increasing β-cell responsiveness and GLP 1 (r=0.7551) and GIP (0.6278) activities. All treatments significantly reduced the glucagon levels (Figure 2b) while the lettuce dietary inclusion produced no significant effect on the incretin hormones, GLP 1 (*P*=0.909) and GIP (*P*=0.990), compared with the untreated insulin-resistant rats. Avicularin treatment slightly but significantly stimulated GLP 1 but not the GIP activities in the insulin-resistant rats. None of the treatments normalized the glucagon, GLP 1, or GIP levels of the insulin-resistant rats.

**Figure 3:**
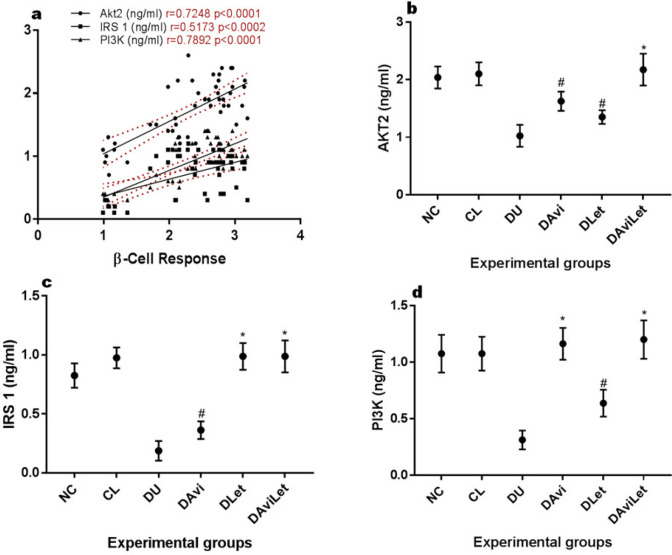
a) Correlation graph of β-Cell response vs Akt2, IRS 1, and PI3K b) mean hepatic Akt2 levels c) mean hepatic IRS 1 levels d) mean hepatic PI3K levels. **- no significant changes relative to normoglycemic rats, #-significantly higher than the control but lower than the negative control (DU), *-no significant difference with the normoglycemic rats

In Figure 3a, a strong positive correlation was found between improved β-cell responsiveness and the insulin signaling factors Akt2 (r=0.7248), IRS 1 (r=0.5173), and PI3K (0.7892). The insulin-resistant rats showed significantly decreased (*P<*0.001) mean Akt2, IRS 1, and PI3K activities. Our findings also indicated that the elevations in Akt2 levels (Figure 3b) in the insulin-resistant rats with the exclusive treatment of either avicularin or lettuce dietary inclusion, was statistically significant. Treatment with combined avicularin and lettuce produced Akt2 levels comparable to the control (*P*=0.728). The result in Figure 3c showed that the insulin-resistant rats treated with the lettuce meal showed hepatic IRS 1 levels comparable (*P*=0.07) to the normoglycemic rats while the PI3K levels (Figure 3d) were significantly lower than the control. Avicularin treatment of the insulin-resistant rats showed slight IRS 1 stimulatory effects but completely restored the PI3K levels (*P*=0.815). The activities of all the insulin signaling molecules in the insulin-resistant rats were restored with the combined treatment of avicularin and lettuce. 

**Figure 4:**
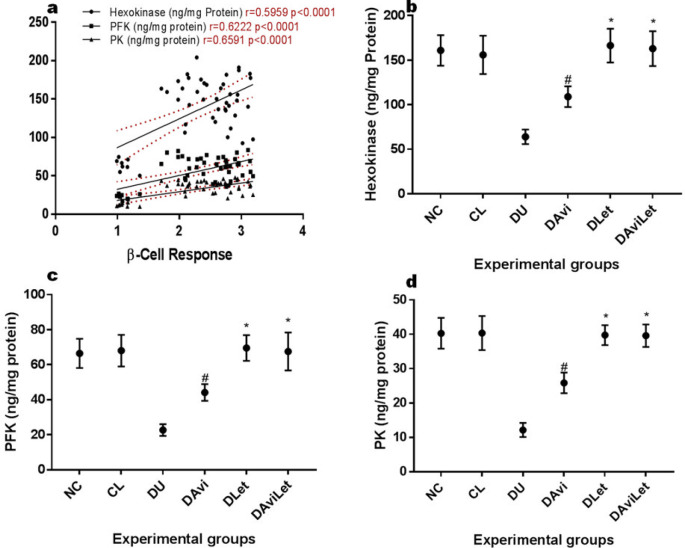
a) Correlation graph of β-Cell response vs hexokinase, phosphofructokinase (PFK), and pyruvate kinase (PK) b) mean hepatic hexokinase levels c) mean hepatic PFK levels d) mean plasma PK levels. #-significantly higher than the control but lower than the negative control (DU), *-no significant difference to the normoglycemic rats

Hexokinase (r=0.5959), PFK (r=0.6222), and PK (r=0.6591) all showed positive correlations with improved β-cell responsiveness (Figure 4a). We observed that the insulin-resistant rats showed significantly lower activities of the kinases compared with the normoglycemic rats. The treatment with exclusive administration of lettuce significantly elevated the activities of hexokinase, PFK, and PK which were not significantly different from the normoglycemic subjects. Avicularin significantly elevated the activities of the kinases but were significantly lower than the control. Restoration of the activities of the glycolytic enzymes in the insulin-resistant rats was achieved with lettuce dietary inclusion and in combination with avicularin.

**Table 1:** HOMA-IR values of study animals before and after induction

**Groups**	**Before induction**	**After induction**
DU	1.7±0.1^*#^	18.9±4.1^*#^
DAvi	1.8±0.2^*#^	21.1±5.7^*#^
DLet	1.7±0.1^*#^	25.9±3.9^*#^
DAviLet	1.6±0.2^*#^	20.3±4.4^*#^

DU- untreated insulin-resistant rats, DAvi- insulin-resistant rats treated with 5000 mg/kg b.w. avicularin, 80% w/w lettuce diet (DLet) or in combination of 5000 mg/kg b.w. avicularin and 80% w/w lettuce diet (DAviLet). *# - no significant difference (*P*>0.05) down the column among study groups

**Table 2:** Biochemical, physiological, and insulin sensitivity parameters of rats

**Groups**	**BMI (g/cm** ^2^ **)**	**FBS** **(mg/dl**	**HbA1c (%)**	**Insulin (µIU/ml)**	**C-peptide (ng/ml)**	**HOMA-IR**	**M0**
NC	0.41±0.03	74.0±4.3	4.6±0.3	10.1±0.7	2.0±0.1	1.8±0.1	2.7±0.2
CL	0.41±0.03	72.1±2.7	4.4±0.3	10.6±1.0	2.0±0.1	1.8±0.1	2.8±0.1
DU**	0.58±0.02	359.8±24.4	10.1±0.7	30.8±3.7	3.9±0.2	27.2±4.8	1.1±0.1
DAvi p values	0.50±0.01^#^	112.5±14.8^#^	4.7±0.2* (0.995)	18.1±1.7^#^	2.9±0.2^#^	4.9±0.8^#^	2.6±0.3^*^ (0.951)
DLet p values	0.42±0.03* (0.827)	120.5±16.3^#^	7.8±0.4^#^	12.7±1.3* (0.078)	2.4±0.1^#^	3.7±0.6* (0.409)	2.0±0.2
DAviLet p values	0.42±0.02* (0.782)	82.1±8.9*	4.5±0.3* (0.999)	11.1±1.1* (0.867)	2.0±0.1* (1.000)	2.2±0.3* (0.998)	2.4±0.3* (0.320)

NC-control group without induced insulin resistance, CL-control rats placed on 80% w/w lettuce diet, DU- untreated insulin-resistant rats, DAvi- insulin-resistant rats treated with 5000 mg/kg BW avicularin, 80% w/w lettuce diet (DLet) or in combination of 5000 mg/kg BW avicularin and 80% w/w lettuce diet (DAviLet). **-significant changes relative to normoglycemic rats, #-significantly higher than the control but lower than the negative control (DU), *-no significant difference to the normoglycemic rats

## Discussion

Notwithstanding the lack of clear evidence in the literature, we however recorded significant anti-obesity effects of lettuce when administered under insulin resistance conditions. Its dietary supplementation with avicularin as an oral insulin-sensitizing agent also potentiated the reversal in weight gain during insulin resistance. Very few studies have linked avicularin to any weight loss effects (46, 47), still, there seems no tangible connection to any anti-obesity effects. Our findings on the glucose-lowering potentials of both avicularin and lettuce agree with the respective reports of Zhu *et al.* (29) and Chadchan *et al* (48). Zhu *et al.* (29) also described the mechanism behind the hypoglycaemic potentials of avicularin. This glucose-lowering effect especially with avicularin led to the complete reversal of glycated hemoglobin to normoglycemic levels. Similar reports elsewhere support our findings showing the insulin-sensitizing effects of lettuce (37, 38), which when combined with avicularin, normalized the c-peptide levels of insulin-resistant rats. Both the decrease in HOMA-IR and consequent elevation in the β-cell response of insulin-resistant rats, on par with normoglycemic rats, further support the antidiabetic theory of the combinatorial administration of avicularin and lettuce dietary inclusion. Evidently, maintaining insulin-resistant subjects on lettuce rich diets with avicularin-based treatment enhances the chances of improving insulin sensitivity. The slight stimulation of GLP 1 as a result of an induced hypoglucagonemia as well as the unresponsive GIP activities observed with the administration of avicularin to insulin-resistant rats, probably eliminates the chances that avicularin achieves its insulin-sensitizing effects via an incretin-like mode of action. At present, no study in the literature supports or contradicts our findings. Rather, the activities of the insulin signaling factors, Akt2, IRS 1, and PI3K under insulin-resistant states offer a better understanding of the insulin-sensitizing effects of lettuce and avicularin. From our findings, during insulin resistance, the secretion of these insulin signaling factors was sub-optimal. In support of this, a study (22) showed decreased secretions of IRS 1 and Akt2 in obese conditions, the major predisposing factor to insulin resistance. According to two studies (49, 50), drugs like liraglutide that up-regulate Akt2 and other key players of the insulin signaling cascade, show potent insulin-sensitizing effects. Thus, lettuce-metformin combination probably up-regulated Akt2 activities that led to the improvement of insulin sensitivity. It was further evident that each of lettuce and avicularin improved the insulin signaling cascade via different targets. Lettuce optimized the hepatic secretion of IRS 1 in the insulin-resistant rats whereas avicularin normalized the activities of hepatic PI3K. The activation of both factors is crucial for effective insulin transduction making them sensitive targets for antidiabetic drugs (51-54). Apparently, with a compromised insulin signaling cascade during insulin resistance, glycolysis also becomes compromised. It was however unclear in our study if the lettuce-induced potentiation of these glycolytic enzymes was by direct stimulatory action or via a compensatory mechanism. Avicularin was less potent than lettuce in reversing the suppression of the glycolysis regulatory enzymes, but when combined administration of lettuce and metformin was initiated, the glycolytic enzymes returned to optimal activities. With our findings, we suggest that the development of a pharmacologically active substance integrating both avicularin and the active constituent of lettuce would enable a more precise and holistically effective strategy to manage insulin resistance. 

## Conclusion

The dietary management of insulin resistance with lettuce and treatment using avicularin all enhance insulin sensitivity at varying degrees via different modes of action. During insulin resistance, avicularin reverses alterations in HbA1c levels, insulin, and PI3K activities with slight incretin stimulatory effect only on GLP 1. On the other hand, complete restoration of BMI, insulin, HOMA-IR, IRS 1, and the glycolytic kinases was achieved by dietary management with lettuce. Thus, a combined pharmacological and dietary approach with avicularin and lettuce is required for a more effective enhancement of insulin sensitivity.
